# Perceived benefits, costs, and relationships on provincial doctors’ inclination to participate in urban–rural medical consortia in Central China: a social exchange theory perspective

**DOI:** 10.3389/fpubh.2024.1474164

**Published:** 2025-01-07

**Authors:** Bowen Zhang, Liang Ma, Wenjie Ma, Dingding Kang, Yiqing Mao

**Affiliations:** ^1^The Affiliated Cancer Hospital of Zhengzhou University and Henan Cancer Hospital, Zhengzhou, China; ^2^College of Public Health, Zhengzhou University, Zhengzhou, China; ^3^Institute for Hospital Management of Henan Province, Zhengzhou, China

**Keywords:** social exchange theory, perceived benefits, perceived costs, perceived relationships, inclination to participate

## Abstract

**Objective:**

This study aims to investigate the perceived benefits, costs, and relationships that influence doctors’ inclination to participate in urban–rural medical consortia (URMC). Furthermore, the study analyzes how perceived relationships moderate the impact of perceived benefits and costs on the inclination to take part in URMC.

**Methods:**

The study was conducted between September 2022 and June 2023 utilizing an online survey in Henan Province, Central China, which included 749 respondents from provincial hospitals. Chi-square and hierarchical logistic regressions were used to determine the perceived factors of the participants’ inclination.

**Results:**

The study indicated that 85.4% doctors demonstrated a strong inclination to participate. Doctors who perceived significant financial benefits, non-financial benefits, and relationships had a high level of inclination to participate. Doctors who perceived high executive costs and technical costs were less likely to express a high level of inclination to participate. When doctors perceived a strong relationship, the influence of perceived non-financial benefits on their inclination to participate tended to be weaker.

**Conclusion:**

This study enhances the understanding of physicians’ motivations for participating in URMC and may inform program leadership and policymakers concerned with developing or enhancing rural medical services. It is essential for managers to enhance incentive mechanisms, strive to minimize both actual and perceived costs, and facilitate the establishment of high-quality professional relationships between provincial physicians and their counterparts in county-level healthcare institutions.

## Introduction

As healthcare reform in China continues to progress, a medical consortium that functions as an integrated delivery system has emerged to enhance the efficiency of the healthcare delivery system (1). As a type of medical consortium, the urban–rural medical consortia (URMC) policy in China aims to vertically integrate healthcare by collaborating with county and provincial hospitals to improve medical service capacity in rural areas. Doctors from provincial hospitals are the main force in promoting URMC and collaborate with county-level doctors to enhance their diagnostic and therapeutic skills by engaging in activities such as case discussions, surgical advice, academic presentations, long-distance consultations, routine outpatient services, and so forth. Current research on medical consortia is bifurcated into the analysis of program outcomes and the optimization of program efficacy, with the former including the impact on health services (2), the impact on healthcare spending (3), and the impact on patient health outcomes (4), and the latter including the enhancement of institutional cooperation based on patient preferences (5), the development of information technology to promote institutional integration (6), and the enhancement of participant perceptions (7). However, no studies have focused on the issue of participants’ inclination to participate. In fact, the inclination of provincial doctors to participate directly determines the operational effectiveness of URMC. Understanding the inclination of physicians to participate and taking steps to increase their inclination to participate is therefore key to ensuring the sustainability of URMC. However, since the year 2017, no studies have been conducted to address this particular issue. It remains unclear whether provincial doctors would be open to participating, as well as what measures could be taken to encourage their involvement in order to guarantee the success and longevity of URMC.

Social exchange theory (SET) ranks as one of the most influential theories in the social sciences due to its versatile framework for comprehending how two parties establish a social connection through reciprocal resource exchanges in repeated interactions (8). SET views interaction behaviors from a benefit/cost perspective. However, this kind of interaction relationship is not a complete economic exchange, but a social relationship, so its benefits and costs could be invisible feelings, such as respect, honor, and effort (9, 10). The reasons for the formation of individual behavioral or inclinations are complex, SET explains the formation logic of individual behavior or inclinations from the perspective of costs and benefits, which provides an effective framework for analyzing the reasons for the formation of this situation and has been successfully used in previous studies, such as exploring the impact of patient gifts (benefits) on doctors’ online service behavior (11), the impact of disclosing personal data (costs) on the consumers’ willingness to shop online (12), etc. Therefore, it is feasible and scientifically sound for this study to analyze the reasons for provincial doctors’ inclinations to participate in URMC based on SET, and at the same time, it can provide strategic recommendations for guiding the participation inclinations of provincial doctors to ensure the sustainability of URMC program.

### Benefit and cost factors in SET

According to SET, the relationship between benefits and costs is an important factor in determining the beginning, maintenance, transformation, or termination of a certain behavior. The beginning or maintenance of exchange behavior is decided by perceiving more benefits than costs; otherwise, when the perceived benefits are less than costs, people’s exchange behavior tends to change or terminate. This viewpoint has been validated in numerous studies. SET provides a framework for deciphering the rules and norms that shape individual transactions and resource exchanges reflected in relationship behaviors through benefit and cost factors (13). In terms of benefits, these encompassed both financial and non-financial aspects. Previous research has suggested that economic incentives and economic returns could directly positively affect an individual’s inclination or behavior (14, 15). Furthermore, prior studies have proposed that non-financial beneficial factors, such as position promotion, social support, reputation, attention, skill improvement, and enjoyment in helping others, positively influence one’s intention, inclination, or behavior (14–20). Regarding cost considerations, previous studies have categorized them into executive costs and technical costs. The execution cost refers to the time, energy, and effort required to complete this exchange behavior, while the technical cost pertains to the loss of technical aspects associated with completing this exchange behavior, such as loss of knowledge rights and leakage of private data. In previous studies, these cost factors have been shown to negatively affect individual intentions and impede the occurrence and continuity of the behavior (14, 16, 18–20). This is consistent with the findings in the China scenario. Therefore, the research hypotheses are as follows:

*H1*: Perceived financial benefits are positively associated with inclination to participate.

*H2*: Perceived non-financial benefits are positively associated with inclination to participate.

*H3*: Perceived executive costs are negatively associated with inclination to participate.

*H4*: Perceived technical costs are negatively associated with inclination to participate.

### Relationship factors in SET

Relationships that exist between partners can be characterized as inter- or intra-organizational associations (9, 13). Previous research has reached a consensus on the impact of relationship factors on exchange behaviors. Different interaction relationships could lead to different behavioral outcomes. This finding is also applicable to the research field of healthcare. For instance, when physicians experience a strong relationship of trust with their organization, their susceptibility to burnout is mitigated (21). Similarly, when patients maintain positive interpersonal relationships with their physicians, they demonstrate a greater inclination to accept vaccinations (22). Previous research has demonstrated that positive social relationships exert a beneficial influence on behavioral outcomes, intentions or inclination (23, 24). This finding may be attributed to the fact that favorable interpersonal relationships facilitate the creation of a relaxed environment and a pleasant mood. Therefore, the research hypothesis is proposed:

*H5*: Perceived relationship is positively associated with inclination to participate.

### The moderating role of relationship factors in SET

SET posits that people form and maintain interpersonal relationships through the exchange of costs and benefits (25). As past experiences shape cost–benefit exchanges, these exchanges, in turn, shape ongoing relationship expectations and actions (23). Therefore, the quality of interpersonal relationships is not only the result of previous social exchange behavior, but also the cause of the next stage of exchange behavior. This forms a behavioral explanation mechanism in SET, where benefit and cost factors can directly affect individual exchange behavior or influence the next stage of behavior by influencing the relationship between exchange parties (26). According to previous research, it has been known that benefit factors have a positive impact on behavior, while cost factors have a negative impact on behavior. However, in incomplete profit-seeking behavior, good interpersonal relationships can weaken the influence of benefit and cost factors on behavior (14). In other words, based on the assumption of non-completely rational individuals from SET, interpersonal relationships can mitigate the positive influence of interest factors on individual behavior, thereby preventing individuals from excessively pursuing interests in exchange behavior. Additionally, these relationships can attenuate the negative influence of cost factors on individual behavior, thus reducing the extent to which individuals are constrained by cost considerations that affect their behavior, intentions, or inclinations. Previous studies have corroborated that relational factors play a negatively moderating role in the benefits and costs of individual exchange behavior or intention. For instance, researchers have found that perceived relationship support moderated the effect of the cost factor (effort) on perceived social benefits and reciprocated behavior (27), and communication relationships moderated the relationship between benefit reciprocity and supply behavior (28). This outcome may be attributed to individuals placing greater emphasis on trust, friendship, or a sense of social obligation in interpersonal relationships. Particularly in the Chinese cultural context, where interpersonal relationships are highly valued, the moderating effect of relational factors may be more pronounced. Therefore, the following hypotheses are then presented.

*H6*: The degree of perceived relationship negatively moderates the impact of doctors’ perceived financial benefits on inclination to participate.

*H7*: The degree of perceived relationship negatively moderates the impact of doctors’ perceived non-financial benefits on inclination to participate.

*H8*: The degree of perceived relationship negatively moderates the impact of doctors’ perceived executive costs on inclination to participate.

*H9*: The degree of perceived relationship negatively moderates the impact of doctors’ perceived technical costs on inclination to participate.

### Research model

**Figure 1:**
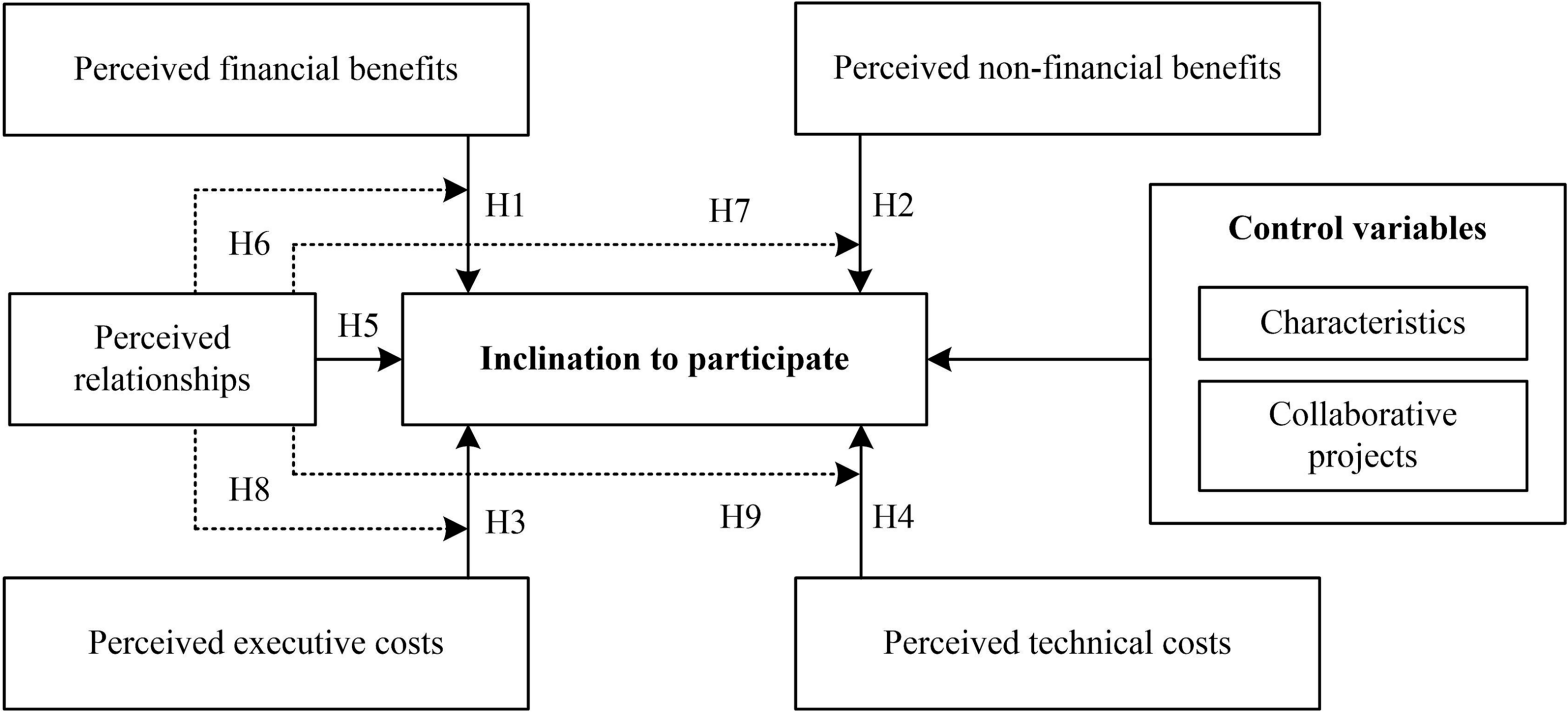
The structural model based on SET.

This study centers on a model that explores the interplay between perceived benefits, perceived costs, and perceived relationships in relation to inclination to participate. According to SET and literatures, perceived benefits and perceived relationships positive influence on inclination to participate, perceived costs negative influence on inclination to participate, and perceived relationships negatively moderate the influence of perceived benefits and costs on inclination level. Finally, the additional factors of inclination to participate—characteristics and collaborative projects—are incorporated as control variables, since it is likely that these also influence the inclination level. This leads to the structural model shown in Figure 1.

## Methods

### Sample population

A cross-sectional survey was carried out between September 2022 and June 2023 in China. The research instrument design process comprised several steps: Initially, based on a comprehensive literature review, the research team developed an interview protocol to engage program administrators and physicians in determining the benefit and cost factors associated with URMC participation. Subsequently, a questionnaire was independently constructed, utilizing a scale-based format for the inquiries. A pilot study was then conducted at a provincial hospital to assess the questionnaire’s reliability and validity, followed by expert consultation to refine the instrument. These modifications culminated in the finalization of the questionnaire. The survey was administered as a web-based questionnaire, with respondents providing informed consent prior to participation.

The URMC project encompasses provincial and county hospitals. The selection of survey respondents in this study is predicated on two primary factors to ensure adequate sample size and representativeness. Firstly, sample county hospitals were selected based on geographical distribution within Henan Province; four county hospitals in the central region and three county hospitals in each of the east, south, west, and north regions were chosen. Secondly, four provincial hospitals in the central region and two provincial hospitals in the northern region of Henan Province were selected based on their collaboration with the chosen county hospitals. The subjects of this study were provincial hospital physicians and therefore, a total of 749 respondents from these hospitals who participated in URMC completed the survey.

### Data measures

#### Dependent variable

The level of inclination to participate in URMC was assessed using a single item, which asked, “I am willing to continue participating in URMC in the future.” Participants were able to indicate their perception level by selecting from the options of “low, ““middle,” “high,” or “I do not know.” This study divided “low” and “I do not know” into “low level of inclination to participate” and others into “high level of inclination to participate.”

#### Independent variables

Based on the literature study, the research team used interviews to identify the benefit and cost factors involved in the URMC program, as shown in Table 1. The independent variables included perceived financial benefits, perceived non-financial benefits, perceived executive costs, perceived technical costs and perceived relationships. Financial benefits included economic returns and rewards. The following non-financial benefits were identified: (1) advancement in position, (2) elevation in professional title, (3) increased recognition, (4) gaining respect, (5) enhanced professional standing, and (6) honing of professional abilities. The participants were requested to assess their perception level using a scale of 0 to 3, with choices spanning from “no” to “high.” The final score was determined by aggregating the scores for each item. The average scores were subsequently categorized into three groups: high, moderate, and low. This study found that the perceived financial and non-financial benefits exhibited strong internal consistency (Cronbach’s α =0.724 and 0.787, respectively). The level of perceived executive costs was measured using four items (18): (1) hardly have time to participate, (2) waste a lot of time, (3) require a lot of effort, and (4) affects normal work. The level of perceived technical costs was measured using two items (18): losing special value and superiority. Perceived relationships was measured using six items based on previous study (29): (1) professional exchanges, (2) cooperation goals, (3) similar vision, (4) communication, (5) familiarity, and (6) trust. The participants were asked to indicate their level of agreement on a five-point Likert scale ranging from 1 (strongly disagree) to 5 (strongly agree). The total score was determined by adding the scores for each item. The average scores were then categorized into three groups: high, moderate, and low. In this study, the internal consistency of perceived executive costs, technical costs, and relationships was found to be satisfactory (Cronbach’s α =0.901, 0.793, and 0.948).

**Table 1:** Contents and measure items of independent variables.

Independent variables	Contents	Items
Perceived financial benefits	Doctors will receive a fixed amount of cash returns (each participation) or cash rewards (more frequently participation) in URMC.	(1) Economic returns
		(2) Rewards
Perceived non-financial benefits	(1) Priority should be given to doctors who have participated in medical consortium when there are opportunities for position and title promotion. (2) Doctors will improve reputation, obtain respect, enhance professional status and skills due to help county-level doctors by participating in medical consortium.	(1) Advancement in position
		(2) Elevation in professional title
		(3) Increased recognition
		(4) Gaining respect
		(5) Enhanced professional standing
		(6) Honing of professional abilities
Perceived execution costs	Doctors needs to invest effort, energy, and time because of participating in the medical consortium.	(1) Hardly have time to participate
		(2) Waste a lot of time
		(3) Require a lot of effort
		(4) Affects normal work
Perceived technical costs	Doctors could lose special value and superiority because of teaching their medical skills to others through participating in URMC.	(1) Losing special value
		(2) Losing superiority
Perceived relationships	A cooperative relationship has been established between provincial doctors and county-level doctors through participating in URMC.	(1) Professional exchanges
		(2) Cooperation goals
		(3) Similar vision
		(4) Communication
		(5) Familiarity
		(6) Trust

#### Control variables

The control variables in this study comprised factors that could potentially influence the dependent variable based on previous research, including individual characteristics such as gender, age, title, and education status (11, 14). Concurrently, physicians’ involvement in various program components may influence their inclination of engaging in URMC. Consequently, this study incorporated the program components in which doctors participate as control variables. These components included case discussions, surgical consultations, academic presentations, continuing education, remote consultation, routine outpatient services, two-way referral (referral for treatment between provincial and county-level hospitals based on the patient’s disease status), scientific research cooperation and medical management.

### Statistical analysis

All statistical analyses were conducted using the most recent version of SPSS (26.0). The chi-square test was used to determine the relationship between the dependent and independent variables. The logistic regression model included only variables with statistically significant differences. The threshold for statistical significance was set at *p* < 0.05 (one-tailed). In order to analyze the influence of perceived financial benefits, non-financial benefits, executive costs, and technical costs on an individual’s inclination to participate in URMC, as well as the moderating effect of perceived relationships, hierarchical logistic regression analysis was conducted (15). The study first decentralized the independent variables and assessed the variance inflation factors (VIFs) among the independent variables to ensure that multicollinearity would not skew the results. Fortunately, no significant multicollinearity was detected among the independent variables (all VIFs ≤2).

## Results

### Sample characteristics

Table 2 showcases the traits of the 749 participants. The percentage of male participants (52.6%) exceeded that of female participants (47.4%). The majority of participants were between 30 and 49 years old (94.4%), held middle and high professional title (86.6%), and possessed master’s or PhD’s degree (92.1%). Surgical medicine was the most common area of specialization for doctors (45.4%). In provincial hospitals, the majority of participants earned more than 10,000 RMB per month (87.9%). In terms of collaborative projects in which they participated, case discussions (78.2%), academic lectures (57.1%), continuing education (74.8%), regular outpatient services (73.6%), and two-way referrals were carried out in more than half of physicians.

**Table 2:** Correlation between dependent variable and independent variables.

Variables	All participants (%)	Inclination to participate (%)		χ^2^ (*p*-values)
		Low	High	
Total participants	*n* = 749 (100)	*n* = 109 (14.6)	*n* = 640 (85.4)	
Demographic characteristics				
Gender				0.578 (0.447)
Male	394 (52.6)	61 (15.5)	333 (84.5)	
Female	355 (47.4)	48 (13.5)	307 (86.5)	
Age				3.717 (0.294)
18–29	26 (3.5)	4 (15.4)	22 (84.6)	
30–39	519 (69.3)	74 (14.3)	445 (85.7)	
40–49	188 (25.1)	26 (13.8)	162 (86.2)	
≥50	16 (2.1)	5 (31.3)	11 (68.8)	
Work year				3.234
≤5	163 (21.8)	25 (15.3)	138 (84.7)	(0.357)
6–10	294 (39.3)	47 (16.0)	247 (84.0)	
10–15	196 (26.2)	21 (10.7)	175 (89.3)	
≥16	96 (12.8)	16 (16.7)	80 (83.3)	
Title				0.260
Primary title	100 (13.4)	15 (15.0)	85 (85.0)	(0.878)
Middle title	541 (72.2)	80 (14.8)	461 (85.2)	
High title	108 (14.4)	14 (13.0)	94 (87.0)	
Education status				0.915
Bachelor	86 (11.5)	15 (17.4)	71 (82.6)	(0.633)
Master	552 (73.3)	80 (14.5)	472 (85.5)	
PhD	111 (18.8)	14 (12.6)	97 (87.4)	
Occupation category				6.218
Surgery medicine	340 (45.4)	58 (17.1)	282 (82.9)	(0.101)
Internal medicine	190 (25.4)	20 (10.5)	170 (89.5)	
Medical technology	182 (24.3)	23 (12.6)	159 (87.4)	
Traditional Chinese medicine	37 (4.9)	8 (21.6)	29 (78.4)	
Monthly income (RMB)				0.347
≤10,000	91 (12.1)	15 (16.5)	76 (83.5)	(0.841)
10,001–20,000	295 (39.4)	43 (14.6)	252 (85.4)	
≥2,0001	363 (48.5)	51 (14.0)	312 (86.0)	
Collaborative projects				
Cases discussion				9.508
No participation	163 (21.8)	36 (22.1)	127 (77.9)	(0.002)
Participated	586 (78.2)	73 (12.5)	513 (87.5)	
Surgical guidance				9.968
No participation	538 (71.8)	92 (17.1)	446 (82.9)	(0.002)
Participated	211 (28.2)	17 (8.1)	194 (91.9)	
Academic lectures				6.617
No participation	321 (42.9)	59 (18.4)	262 (81.6)	(0.010)
Participated	428 (57.1)	50 (11.7)	378 (88.3)	
Continuing education				5.131
No participation	189 (25.2)	37 (19.6)	152 (80.4)	(0.024)
Participated	560 (74.8)	72 (12.9)	488 (87.1)	
Remote consultation				9.861
No participation	623 (83.2)	102 (16.4)	521 (83.6)	(0.002)
Participated	126 (16.8)	7 (5.6)	119 (94.4)	
Regular outpatient services				2.112
No participation	198 (26.4)	35 (17.7)	163 (82.3)	(0.146)
Participated	551 (73.6)	74 (13.4)	477 (86.6)	
Two-way referral				4.558
No participation	369 (49.3)	64 (17.3)	305 (82.7)	(0.033)
Participated	380 (50.7)	45 (11.8)	335 (88.2)	
Scientific research cooperation				16.573
No participation	600 (80.1)	103 (17.2)	497 (82.8)	(< 0.001)
Participated	149 (19.9)	6 (4.0)	143 (96.0)	
Medical management				22.618
No participation	481 (64.2)	92 (19.1)	389 (80.9)	(< 0.001)
Participated	268 (35.8)	17 (6.3)	251 (93.7)	
Independent variables				
Perceived financial benefits				31.020
Low level	120 (16.0)	36 (30.0)	84 (70.0)	(< 0.001)
Middle level	273 (36.4)	40 (14.7)	233 (85.3)	
High level	356 (47.5)	33 (9.3)	323 (90.7)	
Perceived non-financial benefits				31.032
Low level	112 (15.0)	28 (25.0)	84 (75.0)	(< 0.001)
Middle level	439 (58.6)	74 (16.9)	365 (83.1)	
High level	198 (26.4)	7 (3.5)	191 (96.5)	
Perceived execution costs				17.631
Low level	142 (19.0)	8 (5.6)	134 (94.4)	(< 0.001)
Middle level	470 (62.8)	69 (14.7)	401 (85.3)	
High level	137 (18.3)	32 (23.4)	105 (76.6)	
Perceived technical costs				10.704
Low level	204 (27.2)	18 (8.8)	186 (91.2)	(0.005)
Middle level	410 (54.7)	62 (15.1)	348 (84.9)	
High level	135 (18.0)	29 (21.5)	106 (78.5)	
Perceived relationships				42.189
Low level	166 (22.2)	50 (30.1)	116 (69.9)	(< 0.001)
Middle level	283 (37.8)	32 (11.3)	251 (88.7)	
High level	300 (40.1)	27 (9.0)	273 (91.0)	

### Descriptive statistics of independent and dependent variables

In this study, independent variables included perceived financial benefits, perceived non-financial benefits, perceived executive costs, perceived technical costs, and perceived relationships. 47.5% of participants reported a high level of perceived financial benefits, while 40.1% of participants reported a high level of perceived relationships. In addition, more than half of participants reported a middle level of perceived non-financial benefits (58.6%), executive costs (62.8%), and technical costs (54.7%). The detailed results are presented in Table 2.

Among the 749 individuals surveyed, 640 (85.4%) demonstrated a strong inclination to participate in URMC, while 109 (14.6%) showed a low inclination. As illustrated in Table 2, there was a significant contrast between the levels of inclination to participate across the independent variables, including perceived financial benefits (χ^2^ = 31.020, *p* < 0.001), perceived non-financial benefits (χ^2^ = 31.032, *p* < 0.001), perceived executive costs (χ^2^ = 17.631, *p* < 0.001), perceived technical costs (χ^2^ = 10.704, *p* < 0.001), and perceived relationships (χ^2^ = 42.189, *p* < 0.001).

### Predictors and interaction terms affecting the dependent variable

Based on the results of the chi-square test, it was determined that eight control variables exhibited statistically significant correlations with an individual’s inclination to engage in URMC, encompassing participation in case discussions, surgical guidance, academic lectures, continuing education, remote consultation, two-way referral, scientific research collaboration, and medical management. To investigate the predictors of inclination to participate, hierarchical logistic regression analysis was conducted, while keeping these variables under control. Model 1 only contains control variables, and the results show that doctors who participated in medical management had a high level of inclination to participate (*β* = 0.892, *p* = 0.002). Model 2 added the influence of doctors’ perceived financial benefits, non-financial benefits, executive costs, technical costs and relationships on the inclination to participate based on Model 1. According to the results, doctors who had a high level of perceived financial benefits (*β* = 0.450, *p* = 0.007), non-financial benefits (*β* = 0.689, *p* = 0.002), and relationships (*β* = 0.765, *p* < 0.001) had a high level of inclination to participate. Doctors who had a high level of perceived executive costs (*β* = −0.751, *p* = 0.001) and technical costs (*β* = −0.496, *p* = 0.004) had a low level of inclination to participate. Thus, H1~H5 were supported.

**Figure 2:**
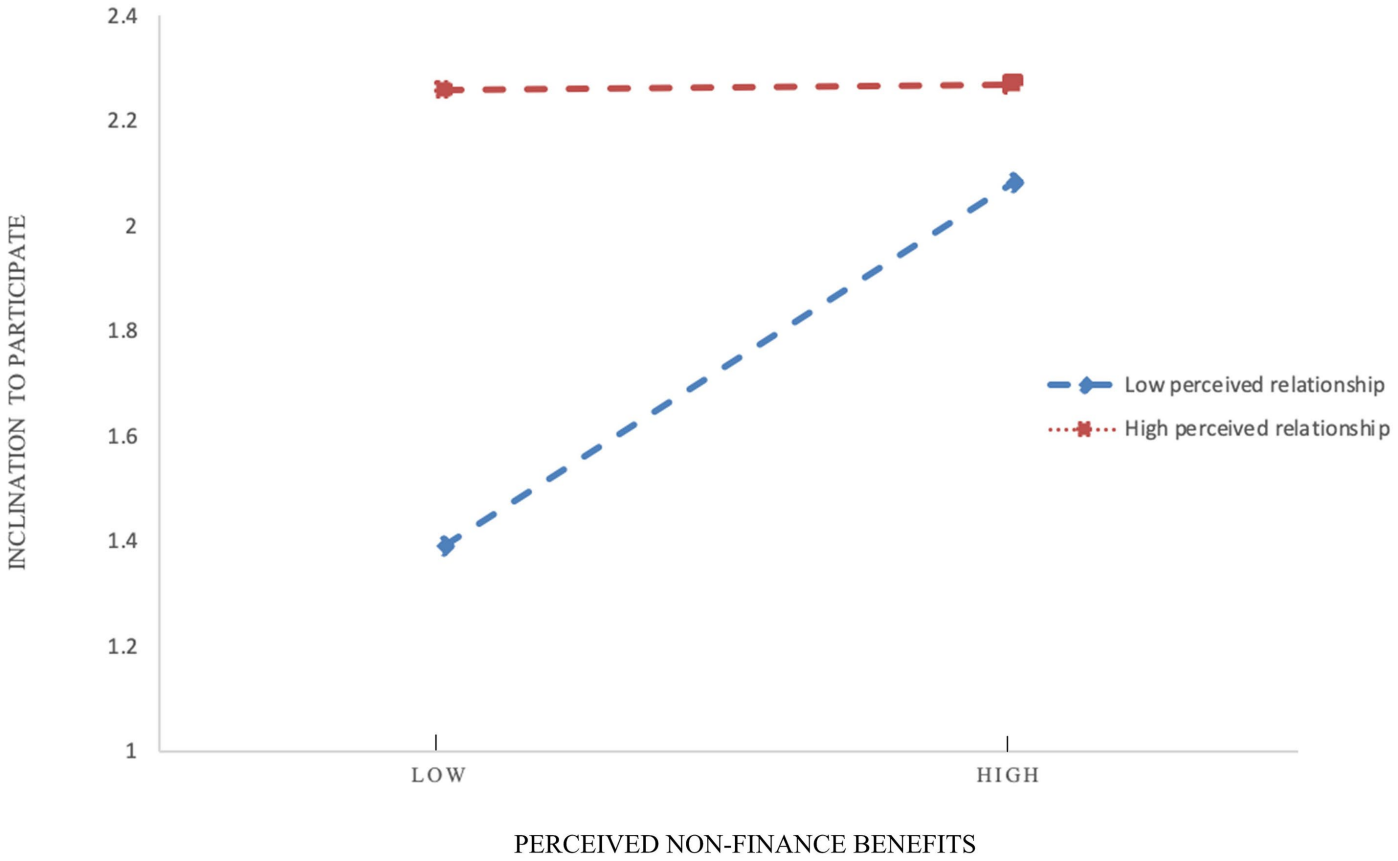
Interaction between perceived non-financial benefits and relationships.

Finally, this study analyzed the effects of the interaction terms. Model 4 showed that when doctors had a high level of perceived relationships, the degree of influence perceived non-financial benefits on inclination to participate in URMC could be weakened (*β* = −0.618, *p* = 0.018). Thus, H7 was supported. The interaction between perceived non-financial benefits and relationships was shown in Figure 2. First, when the degree of perceived non-financial benefits increases, doctors’ inclination to participate increases, and the trends of low and high level of relationship perception were the same. Second, as the degree of relationship perception increases, the influence of non-financial benefit perception on participation inclination weakens, indicating that doctor perceived relationships negatively moderated the relationships between non-financial benefit perception and inclination to participate in URMC. In addition, the study showed that perceived relationship did not play a moderating role in the effect of financial benefits, execution costs, and technology costs on physicians’ inclination to participate. Therefore, hypotheses H6, H8, and H9 were not supported. The results are presented in Table 3.

**Table 3:** Logistic regression analysis.

Variables	Dependent variable: inclination to participate					
	Model 1	Model 2	Model 3	Model 4	Model 5	Model 6
Control variables						
Cases discussion	1.551 (0.085)	1.872 (0.027)	1.916 (0.024)	2.009 (0.022)	1.891 (0.026)	1.908 (0.025)
Surgical guidance	0.115 (0.711)	0.237 (0.480)	0.261 (0.444)	0.246 (0.474)	0.206 (0.540)	0.239 (0.478)
Academic lectures	0.200 (0.358)	0.098 (0.681)	0.095 (0.692)	0.127 (0.599)	0.086 (0.719)	0.099 (0.677)
Continuing education	−1.157 (0.193)	−1.449 (0.081)	−1.481 (0.075)	−1.589 (0.066)	−1.475 (0.076)	−1.479 (0.077)
Remote consultation	0.126 (0.792)	0.235 (0.648)	0.205 (0.695)	0.207 (0.689)	0.256 (0.618)	0.232 (0.651)
Two-way referral	0.126 (0.569)	0.026 (0.914)	0.010 (0.968)	0.031 (0.899)	0.041 (0.863)	0.008 (0.975)
Scientific research cooperation	0.945 (0.059)	0.757 (0.156)	0.789 (0.145)	0.758 (0.155)	0.763 (0.150)	0.750 (0.159)
Medical management	0.892 (0.002)	0.842 (0.008)	0.859 (0.007)	0.864 (0.007)	0.831 (0.008)	0.848 (0.007)
Independent variables						
Perceived financial benefits	N/A	0.450 (0.007)	0.310 (0.088)	0.479 (0.005)	0.454 (0.006)	0.461 (0.006)
Perceived non-financial benefits	N/A	0.689 (0.002)	0.729 (0.001)	0.503 (0.031)	0.694 (0.002)	0.694 (0.002)
Perceived execution costs	N/A	−0.751 (0.001)	−0.730 (0.001)	−0.741 (0.001)	−0.726 (0.001)	−0.756 (0.001)
Perceived technical costs	N/A	−0.496 (0.004)	−0.533 (0.002)	−0.525 (0.003)	−0.496 (0.004)	−0.438 (0.019)
Perceived relationships	N/A	0.765 (< 0.001)	0.664 (< 0.001)	0.625 (< 0.001)	0.810 (< 0.001)	0.743 (< 0.001)
Perceived financial benefits × Perceived relationships	N/A	N/A	−0.385 (0.048)	N/A	N/A	N/A
Perceived non-financial benefits × Perceived relationships	N/A	N/A	N/A	−0.618 (0.018)	N/A	N/A
Perceived execution costs × Perceived relationships	N/A	N/A	N/A	N/A	−0.304 (0.316)	N/A
Perceived technical costs × Perceived relationships	N/A	N/A	N/A	N/A	N/A	0.176 (0.429)

N/A, not applicable.

## Discussion

The inclination of provincial physicians to participate in URMC has a direct impact on the operational effectiveness of the program. Fortunately, this research demonstrated that the majority of doctors from provincial hospitals who took part in URMC program expressed a strong inclination to continue their involvement in the future. However, still 14.6% of physicians expressed reluctance to continue participation in URMC. Based on the SET theory, this study explored the benefit, cost, and relationship factors that influence the inclination to participate of provincial physicians. This expands the potential applications of SET theory in healthcare, while the findings of the study can provide an empirical foundation for enhancing the inclination of provincial physicians to participate, including optimizing benefit acquisition, regulating cost expenditures, and strengthening relationship networks, thereby ensuring URMC program sustainability. The primary findings of this study are summarized below.

This study showed that physicians with a high level of perceived financial benefits and non-financial benefits had a high level of inclination to participate. This result once again confirms the applicability of SET in the Chinese context and is consistent with previous research findings (11, 14, 20). The acquisition of benefits by the physicians is primarily derived from the incentive measures established by URMC (30). These measures, including the implementation of financial incentives (economic returns and rewards) and non-financial incentives (position and professional title promotion). First, doctors are entitled to receive a cash subsidy for each time they take part in URMC, as stipulated by regulations. They may also be eligible for additional rewards if they have participated more frequently than others. Objective financial income is important to the young and middle-aged population who are the primary labor force of their families. Second, the majority of participants in this study had a middle professional title, hence, they could have a high need to promote title or position. The non-financial incentives of URMC can help them achieve this goal, such as title promotion. Consequently, physicians participating in the URMC program are able to receive appropriate and equitable financial and non-financial benefits that aligns with population expectations and continues to foster physicians’ inclination to participate (31, 32). However, this study showed that 1 6.0% of physicians and 1 5.0% of physicians perceived low levels of financial and non-financial benefits, suggesting that the incentives of the URMC program need to be continually improved in order to ensure that the population is motivated to participate in the program by increasing the overall perceived benefits to physicians (31, 33). Additionally, there are potential benefits that provincial doctors can gain by participating in URMC, such as expanding fame, obtaining respect, and improving professional status, have a great influence on the actions and intentions of young individuals in previous studies (34), also included inclination to participate in this study.

The research revealed that doctors who perceived lower executive and technical costs demonstrated a higher inclination to participate in URMC. This results are consistent with previous findings (20, 35). First, the perceived executive costs of doctors includes the effort, energy, and time required to participate in the medical consortium (18). Owing to the current inverted triangle in the healthcare system in China, provincial hospitals undertake a large amount of medical service work, and the workload of provincial doctors is generally very heavy (36). In this context, doctors of provincial hospitals not only need to complete their originally heavy work tasks, but also need to put in extra effort and time to participate in medical consortium projects, which is a significant executive cost for them. Hence, doctors’ inclination may be reduced if there is a lack of reasonable arrangements (37). Second, the perceived technical costs of doctors refers to losing special value and superiority because of teaching their medical skills to doctors in county-level hospitals by participating in URMC (18). In this survey, 18.0% of the doctors expressed a high level of concern about losing their professional technical value and superiority. According to SET theory, although executive and technical costs exist in URMC, if the physician’s perception of benefits is not less than the perception of costs, then the overall inclination of provincial physicians to participate remains relatively optimistic. However, we cannot ignore the impact of cost factors on the doctors’ inclination to participate. In addition to implementing effective incentive mechanisms, managers of URMC should make every reasonable effort to mitigate the physicians’ actual and perceived costs associated with participation in the process. This may include providing a conducive work environment, offering flexible work arrangements, ensuring regular periods of leave, and promoting cultural education, which will be crucial.

The findings of this study indicated that doctors who perceived strong relationships had a greater readiness to participate in URMC. Meanwhile, as the degree of relationship perception increases, the influence of non-financial benefit perception on participation inclination weakens, indicating that doctor perceived relationships reverse moderated the relationships between non-financial benefit perception and inclination to participate in URMC. Similar to previous research findings (23, 24), this study demonstrates the positive impact of social relationships on individual behavior or intention in the context of incomplete economic exchange. Good relationships between doctors from provincial and county-level hospitals create a relaxed working atmosphere in the medical consortium, which not only helps provincial doctors to complete collaborative work more easily, gain a sense of achievement, but also establish partnerships beyond work relationships. Therefore, good relationships between doctors from provincial and county-level hospitals can help provincial doctors gain some non-financial benefits, such as friendship, thereby regulating the impact of non-financial benefits obtained from hospitals on their inclination to participate in URMC. Investing in building high-quality social relationships appears to be a practical necessity for organizations (38). It is imperative for managers to devote significant attention to organizational design, not only considering traditional incentive mechanisms but also recognizing the increasing significance of cultivating social connections to promote collaborations in a resilient manner (13).

## Limitations

This study encountered certain limitations. First, the perceived benefit and cost levels were determined through self-reporting, which means that the results only reflect the participants’ subjective perspectives on certain variables. It is recommended that future studies incorporate objective data to assess the benefits and costs more accurately. Second, this study only explored the impact of three variables on inclination to participate, and there are more influencing factors that have not been paid attention to according to SET, such as specification, management, and service quality. Future studies should improve the mechanism model of inclination to participate by incorporating additional factors.

## Conclusion

The majority of doctors from provincial hospitals participating in URMC in Central China reported a high level of inclination to continue their involvement, according to this study. This finding is advantageous for the long-term growth of URMC and demonstrates that initiatives to support projects are successful. According to SET, this study demonstrated that doctors who perceived significant financial and non-financial advantages and low levels of executive and technical costs displayed a high level of eagerness to participate. Therefore, the decision-makers at URMC should carefully evaluate and research the efficacy of each type of incentive, and make every reasonable effort to minimize the doctors’ actual and perceived costs associated with participating in the process. This research indicated that doctors who perceived strong relationships had a greater inclination to participate. Meanwhile, as the degree of relationship perception increases, the influence of non-financial benefit perception on participation inclination weakens, indicating that doctors perceived relationships negatively moderated the relationships between non-financial benefit perception and inclination to participate in URMC. It seems important for organizations to invest and design to help provincial doctors build high-quality relationships with doctors of county-level hospitals.

## Data Availability

The raw data supporting the conclusions of this article will be made available by the authors, without undue reservation.
