# Supplementary material for: Perceived benefits, costs, and relationships on provincial doctors’ inclination to participate in urban–rural medical consortia in Central China: a social exchange theory perspective
Source: Front Public Health. 2025 Jan 7;12:1474164. doi: 10.3389/fpubh.2024.1474164 (PMC11756516; doi:10.3389/fpubh.2024.1474164)
Supplement: Supplementary file 1 [file Table_1.DOCX]

**Appendix 1 Questionnaire**

# I. General Information

| 1. What's your gender？ A. male B. female |
| --- |
| 2. How old are you? age |
| 3. How many years have you worked? years |
| 4. What’s your professional title? A. primary title B. middle title C. high title |
| 5. What’s your education level? A. bachelor degree B. master degree C. PhD degree |
| 6. What’s your occupation category?  A. surgical medicine B. internal medical C. medical technology D. traditional Chinese medicine  E. others |
| 7. What’s your monthly income? RMB |
| 8. What hospital do you employ? |

**II．Collaborative contents in urban-rural medical consortia**

| 1. May I ask if you have participated in the case discussions？  A. no participation B. participated |
| --- |
| 2. May I ask if you have participated in the surgical guidance?  A. no participation B. participated |
| 3. May I ask if you have participated in the academic lectures?  A. no participation B. participated |
| 4. May I ask if you have participated in the continuing education?  A. no participation B. participated |
| 5. May I ask if you have participated in the remote consultation?  A. no participation B. participated |
| 6. May I ask if you have participated in the regular outpatient service?  A. no participation B. participated |
| 7. May I ask if you have participated in the two-way referral?  A. no participation. B. participated |
| 8. May I ask if you have participated in the scientific research cooperation?  A. no participation. B. participated |
| 9. May I ask if you have participated in the medical management?  A. no participation. B. participated |
| 10. Have you participated in any other projects within the urban-rural medical consortium besides the aforementioned ones? |

**III. Incentive in urban-rural medical consortia**

| 1. I had economic returns by participating in the urban-rural medical consortium.  A. no economic return B. had a low level of economic returns  C. had a middle level of economic returns D. had a high level of economic returns |
| --- |
| 2. I had finical rewards by participating in the urban-rural medical consortium.  A. no reward B. had a low level of rewards  C. had a middle level of rewards D. had a high level of rewards |
| 3. I had position promotion by participating in the urban-rural medical consortium.  A. no promotion B. had a low level of promoting  C. had a middle level of promoting D. had a high level of promoting |
| 4. I had professional title promotion by participating in the urban-rural medical consortium.  A. no promotion B. had a low level of promoting  C. had a middle level of promoting D. had a high level of promoting |
| 5. I had expanded fame by participating in the urban-rural medical consortium.  A. no expand B. had a low level of expanding  C. had a middle level of expanding D. had a high level of expanding |
| 6. I had obtained respect by participating in the urban-rural medical consortium.  A. no obtain B. had a low level of obtaining  C. had a middle level of obtaining D. had a high level of obtaining |
| 7. I had enhanced professional status by participating in the urban-rural medical consortium.  A. no enhance B. had a low level of enhancing  C. had a middle level of enhancing D. had a high level of enhancing |
| 8. I had improved professional skills by participating in the urban-rural medical consortium.  A. no improve B. had a low level of improving  C. had a middle level of improving D. had a high level of improving |

**IV. Attitude and altruism**

| 1. I think the urban-rural medical consortium is effective in improving the service capacity of county-level hospitals.  A. had a low level of effective B. had a middle level of effective  C. had a high level of effective D. I don’t know |
| --- |
| 2. I am willing to continue participating in the urban-rural medical consortium in the future.  A. have a low level of willingness B. have a middle level of willingness  C. have a high level of willingness D. I don’t know |
| 3. Even if there are no benefits, I am willing to participate in the urban-rural medical consortium.  A. have a low level of willingness B. have a middle level of willingness  C. have a high level of willingness D. I don’t know |
| 4. I may lose my special value in the professional field because of participating in the urban-rural medical consortium.  A. absolutely disagree B. disagree C. generally D. agree E. absolutely agree |
| 5. I may lose the superiority of exclusive technology in the professional field because of participating in the urban-rural medical consortium.  A. absolutely disagree B. disagree C. generally D. agree E. absolutely agree |
| 6. I hardly have time to participate in the urban-rural medical consortium.  A. absolutely disagree B. disagree C. generally D. agree E. absolutely agree |
| 7. It will waste a lot of my time to participate in the urban-rural medical consortium.  A. absolutely disagree B. disagree C. generally D. agree E. absolutely agree |
| 8. It requires a lot of effort to participate in the urban-rural medical consortium.  A. absolutely disagree B. disagree C. generally D. agree E. absolutely agree |
| 9. I am worried that participating in the urban-rural medical consortium may affect my normal work.  A. absolutely disagree B. disagree C. generally D. agree E. absolutely agree |

**V. Collaboration evaluation of urban-rural medical consortia**

| 1. Hospitals have a clear direction for promoting urban-rural medical consortium projects.  A. absolutely disagree B. disagree C. generally D. agree E. absolutely agree |
| --- |
| 2. Hospitals have dedicated leaders in promoting urban-rural medical consortium projects.  A. absolutely disagree B. disagree C. generally D. agree E. absolutely agree |
| 3. I am able to generate technological innovation in participating in urban-rural medical consortium projects.  A. absolutely disagree B. disagree C. generally D. agree E. absolutely agree |
| 4. I often have professional exchanges with my partners of the urban-rural medical consortium.  A. absolutely disagree B. disagree C. generally D. agree E. absolutely agree |
| 5. I have consistent cooperation goals with my partners of the urban-rural medical consortium.  A. absolutely disagree B. disagree C. generally D. agree E. absolutely agree |
| 6. I share a similar vision with my partners of the urban-rural medical consortium.  A. absolutely disagree B. disagree C. generally D. agree E. absolutely agree |
| 7. The urban-rural medical consortium project have clear charters that define the responsibilities and obligations of both parties.  A. absolutely disagree B. disagree C. generally D. agree E. absolutely agree |
| 8. I can communicate information quickly and accurately with my partners of the urban-rural medical consortium.  A. absolutely disagree B. disagree C. generally D. agree E. absolutely agree |
| 9. I know the professional background of my partners of the urban-rural medical consortium.  A. absolutely disagree B. disagree C. generally D. agree E. absolutely agree |
| 10. I trust the abilities and sense of my partners of the urban-rural medical consortium.  A. absolutely disagree B. disagree C. generally D. agree E. absolutely agree |
